# HIV-1 Env Does Not Enable the Development of Protective Broadly Neutralizing Antibodies in an Experimental Autoimmune Encephalomyelitis Mouse Model

**DOI:** 10.3389/fimmu.2021.771359

**Published:** 2021-11-02

**Authors:** Gabriel Siracusano, Annamaria Finardi, Claudia Pastori, Vittorio Martinelli, Roberto Furlan, Lucia Lopalco

**Affiliations:** ^1^ Immunobiology of HIV, Division of Immunology, Transplantation and Infectious Diseases, San Raffaele Scientific Institute, Milan, Italy; ^2^ Clinical Neuroimmunology Unit, Institute of Experimental Neurology (INSpe), Division of Neuroscience, San Raffaele Scientific Institute, Milan, Italy; ^3^ Neurology Department, San Raffaele Scientific Institute, Milan, Italy

**Keywords:** EAE, HIV-1, Env, bNAbs, tolerance

## Abstract

Recent studies showed that immunological tolerance may restrict the development of Env-specific autoreactive broadly neutralizing antibodies. This evidence is consistent with the finding that Env immunization of a systemic lupus erythematosus (SLE) murine model produced antibodies that neutralize tier 2 HIV-1 strains. In this study, we address the possibility of eliciting neutralizing anti-Env antibodies in other autoimmune diseases such as multiple sclerosis (MS). While, as reported for SLE, we showed for the first time that a small number of HIV-1 negative, relapsing remitting MS patients exhibited antibodies with neutralizing properties, our attempts at inducing those antibodies in a EAE mouse model of MS failed. The success in eliciting Env-specific neutralizing antibodies might be related to the specific characteristics of the autoimmune disease, or it might rely in improving the vaccination design. Studies using mouse models are useful to gain insight in how HIV-specific neutralizing antibody responses are regulated in order to develop a protective HIV-1 vaccine.

## Introduction

HIV-1, the causative agent of the acquired immunodeficiency syndrome (AIDS), is a global public health problem accounting for the infection of almost 40 million people worldwide (1). Approximately 10-20% of HIV-1-infected subjects develop potent and specific broadly neutralizing antibodies (bnAbs) able to neutralize the 90% of the circulating HIV-1 strains but only 1-2 years after primary infection. These Abs recognize several conserved and less immunogenic neutralizing epitopes of the HIV-1 envelope (Env) protein complex, including the gp120 CD4 binding site (CD4bs), gp41 membrane-proximal external region (MPER), gp120 V1/V2 loop, gp120 V3-glycans (2, 3). The elicitation of such antibodies is one of the main challenges for the development of HIV-1 vaccine candidates. A subset of bnAbs, directed against CD4bs and MPER, has shown poly/autoreactive properties, including the recognition of self-antigens, specifically phospholipids, dsDNA, ubiquitin ligase E3A and kynureninase enzymes, the SF3B3 splicing factor and histone H2A (2, 4–8). The autoreactive property arose the hypothesis that B cells expressing an autoreactive bnAb could be eliminated by immunological tolerance mechanisms during their maturation process, impairing their development (7, 9). Only the autoreactive B cells with weak autoreactivity escape the negative selection of the central tolerance and enter the peripheral lymphoid compartments of healthy individuals and wild-type mice, and become anergic and short-lived (10). Autoimmune prone mice (11, 12) and autoimmune individuals (13–15) harbor serum polyreactive antibodies recognizing HIV-1 in the absence of infection. Interestingly, a systemic lupus erythematosus (SLE) murine model produced antibodies that neutralize tier 2 HIV-1 strains, strengthening the hypothesis that immunological tolerance indeed limits wild-type B cells from producing Env-specific antibodies (10). Therefore, breaching the immunological tolerance might be a strategy to successfully develop those antibodies. Moreover, interesting evidences come from epidemiologic studies the frequency of HIV-1 infection in subjects affected SLE is very low (13, 16–18), and a SLE patient harbored plasma able to neutralize a wide breadth of HIV-1 strains and to control HIV-1 infection in the absence of antiretroviral therapy (5).

In this study, we wondered whether the elicitation of neutralizing anti Env antibodies was restricted to SLE or could be observed in other autoimmune diseases such as multiple sclerosis (MS). Interestingly, as reported for SLE, HIV-1-infected subjects have a lower risk to develop MS compared to that of the general population (10), and very few cases of comorbidity between MS and HIV-1 infection have been reported (10, 13, 16–21). On this basis, we also attempted at verifying whether the development of bnAbs in mice had potential effect on the onset or the clinical course of the experimental autoimmune encephalomyelitis (EAE), the most well studied mouse model of MS.

## Material and Methods

### Patient Selection and Data Collection

A group of 40 patients diagnosed with relapsing-remitting multiple sclerosis (MS-RR), 6 with primary progressive multiple sclerosis (MS-PP), 5 with secondary progressive multiple sclerosis (MS-SP), 10 with clinically isolated syndrome (CIS) and 10 with other neurological disease (OND) participated in the study (**Table 1**). Nine sera derived from healthy donors were used as negative controls. The patients provided their written informed consent to participate in this study. All groups were randomly selected and consisted of age- and sex-matched, HIV-1 negative patients.

**Table 1:** Demographic and clinic characteristics of MS patients.

Subjects	Mean age (range)	Sex (F/M)	Mean EDSS
CIS (N=10)	36 (21-53)	6/4	1.0 ± 1.0
MS-RR (N=40)	38 (23-57)	22/18	1.5 ± 1.0
MS-PP (N=6)	48 (25-59)	3/3	3.5 ± 1.4
MS-SP (N=5)	54 (46-64)	4/1	2.9 ± 1.3
OND (N=10)	68 (60-77)	3/7	n.a.

CIS, Clinically isolated syndrome; MS, multiple sclerosis; RR, relapsing-remitting; PP, primary progressive; SP, secondary progressive; OND, other neurological disease; EDSS, expanded disability status scale. n.a., not applicable.

### Immunoglobulin G (IgG) Purification From Sera of Patients

IgG were purified from the sera of patients by HiTrap Protein G HP column according to the manufacturer’s instructions. The volume of the purified IgG from each sample was measured and the dilution factor with respect to the purified serum starting volume was calculated. The purified IgG were then tested in TZM-bl neutralization assays.

### Viral Stock Production and Titration

Two HIV-1 pseudoviruses, tier 1 and tier 2 virus strains, respectively with a higher and lower level of neutralization susceptibility were employed in the TZM-bl assay: SF162 (Clade B, Tier 1 virus), QH0692 (Clade B, Tier 2). Viral stocks were generated and titered. In particular, 293 T cell-derived stocks of pseudoviruses were generated by proviral DNA transfection using FuGENE HD, according to the manufacturer’s protocol (Promega, Madison, WI). Viral supernatants were harvested 72 h post transfection and clarified at 1800 rpm for 20 min. The virus stocks were analysed for firefly luciferase expression in the TZM-bl cell line. Four replicates of five-fold dilutions of viruses were added to 96 flat-bottomed plate wells containing 10^4^ TZM-bl cells per well, in DMEM supplemented with 10% FBS and 7.5 μg/ml DEAE-dextran in a final volume of 200 μl. After 48 h of incubation at 37°C, 100 μL of culture medium were removed from each well and replaced with 100 μL of Bright Glo luciferase reagent (Promega). After 2 min of incubation, 100 μL of the cell lysate were transferred to a 96 well white solid plate and luminescence was measured using a Victor Light 2030 luminometer (Perkin Elmer). Neutralization titers were expressed as ID50 values, defined as the virus dilution required to achieve half maximal infection (Reed–Muench calculation).

### Neutralization Assays

In order to assess the neutralizing activity of human and mice samples, the infectivity reduction was measured as a reduction in Luc reporter gene expression after a single round of virus infection in TZM-bl cells with pseudotyped viruses. In order to demonstrate the specificity of HIV neutralization, an HIV-unrelated virus (VSV-G virus, strain SVA.MLV#922) was also included. Briefly, 200 TCID50 of pseudoviruses in 50 μL of culture media were incubated with 100 μL of two different dilutions of sera (1:20 and 1:60) by using D-MEM with 10% FBS in a 96-well plate for 1h at 37°C. A 100 μL solution of TZM-bl cells (10^4^ cells per well) containing 18.75 μg/ml DEAE dextran was added (final concentration 7.5 μg/ml); the cultures were then incubated at 37°C for 48h. Assay controls included replicate wells of TZM-bl cells alone (cell control) and TZM-bl cells with virus (virus control). Two replicates were performed per each point. Percentage of neutralization (% NT) was expressed as reduction in relative luminescence units (RLU) compared to the virus control wells, after subtraction of cell control luminescence.

### Experimental Autoimmune Encephalomyelitis (EAE) Induction

Seven-week and 28 weeks-old C57BL/6 female mice were purchased from Charles River. All mice were housed in specific-pathogen-free conditions, in roomy cages, allowing free access to food and water with a constant light/dark cycle. All efforts were made to minimize animal suffering and to reduce the number of mice used, in accordance with the European Communities Council Directive of November 24, 1986 (86/609/EEC). All procedures involving animals were performed according to the animal protocol guidelines prescribed by Institutional Animal Care and Use Committee (IACUC) at San Raffaele Scientific Institute (Milan, Italy). Mice were immunized by subcutaneous injections with 100μl of 2 mg/ml Myelin Oligodendrocyte Glycoprotein (MOG)35–55 (from Multiple Peptide System) in incomplete Complete Freund’s Adjuvant (CFA) containing 8 mg/ml Mycobacterium tuberculosis (strain H37Ra; Difco). Pertussis toxin (Sigma) (500 ng/mice) was injected on the day of the immunization and again two days later. EAE was successfully induced in 100% of mice, although with sign severity and onset variability.

### Clinical Score and Weight

Mice were weighed and scored for clinical signs daily up to the day of culling. Clinical assessment of EAE was performed in a blinded fashion according to the following scoring criteria: 0 = healthy, 1 = limp tail, 2 = ataxia and/or paresis of hindlimbs, 3 = paralysis of hindlimbs and/or paresis of forelimbs, 4 = tetraparalysis, and 5 = moribund or death.

### Mice Immunization and Antibody Collection From Sera

Four different adjuvants were compared in the study; Freund’s adjuvant, aluminum hydroxide, RIBI, and Montanide ISA 720 were added to immunogen preparations. All adjuvants were provided by Sigma-Aldrich. Mice were immunized i.p. with 50 μg/mice of trimeric gp140 (CN54). Blood samples from immunized mice were collected from the eye before immunization (T0) and 7 (T1) and 14 (T2) days after immunization, centrifuged at 1500 rpm to recover plasma, heat inactivated at 56°C for 30 minutes, and stored at -20°C before analysis.

### Determination of Total (i) and gp140-specific (ii) IgG by Enzyme-Linked Immunosorbent Assay (ELISA)

(i) Total IgG ELISA. To quantify serum immunoglobulins, Maxisorp Immunoplates (Thermo Scientific) were coated with 0,1 μg/well with Goat anti-mouse IgA + IgG + IgM (H+L) antibody (Kpl-SeraCare) in PBS for 1 h at 37°C. The plates were saturated for 1 h with 1% skim milk powder (Sigma-Aldrich) in PBS. Commercial preparation of mouse IgG (Sigma- Aldrich) was used as standard at concentrations ranging from 4 to 0.06 μg/ml (2-fold serial dilutions) to generate a calibration curve. Duplicate 2-fold dilutions of heat-inactivated sera starting from 1:1000 and standard were incubated 1 h at 37°C. Then, 1:5000 peroxidase-conjugated goat anti-mouse IgG (Sigma- Aldrich) was added and incubated for 30 min at 37°C. The enzymatic reaction was developed with the TMB Microwell Peroxidase Substrate System (KPL, Gaithersburg, MD, USA) and read at 492 nm. The IgG concentration of the samples was interpolated from four-parameter standard curve.

(ii) gp140 ELISA. Maxisorp Immunoplates (Thermo Scientific) were coated overnight at 4°C with 0.1 μg/well gp140 (CN54) diluted in 50 mM pH 9.5 carbonate buffer. The following day, both Env-coated and uncoated plates were blocked for 1 h at 37°C with PBS supplemented with 5% skim milk, 5% FBS, and 0.1% Tween 20. Duplicate 2-fold dilutions starting from 1:20 of heat-inactivated sera were added at 50 μl/well and incubated for 1 h at 37°C. The plates were washed and 1:5000 peroxidase-conjugated goat anti-mouse IgG (Sigma- Aldrich) was added for 30 min at 37°C. HIV-1-infected and healthy individuals were used as positive and negative controls, respectively, using 1:2000 biotynylated-goat anti-human-IgG (Southern Biotech) followed by 1:3000 HRP-conjugated streptavidin (Vector). Plates were developed by adding 100 μl/well of TMB and the reaction stopped with 50 μl/well of 10% H2SO4 after 5 minutes. Absorbance was read at 450 nm using a Microplate Reader (Biotek). The absorbance values recorded for uncoated plates were subtracted from those on the Env-coated plates. Cut-offs for each sample dilution was subsequently determined by calculating the mean “adjusted” absorbance + 3 SD for negative controls. The reciprocal endpoint titer of antibody in sera was defined as the last sample dilution that produced an absorbance greater than the cut-off.

### Statistical Analysis

The two-tailed Student or Mann–Whitney and Kruskal–Wallis test were used to compare the results obtained by different groups of animals. Kaplan–Meier survival analysis was conducted to compare animal groups for their effectiveness in predicting clinical outcomes. The level of significance was considered at p=0.05. GraphPad Prism 8.2.0 (GraphPad Software, Inc., San Diego, CA, USA) was used to perform statistical analysis.

## Results

### A Small Fraction of MS Patients Harbour Neutralizing Abs

**Figure 1:**
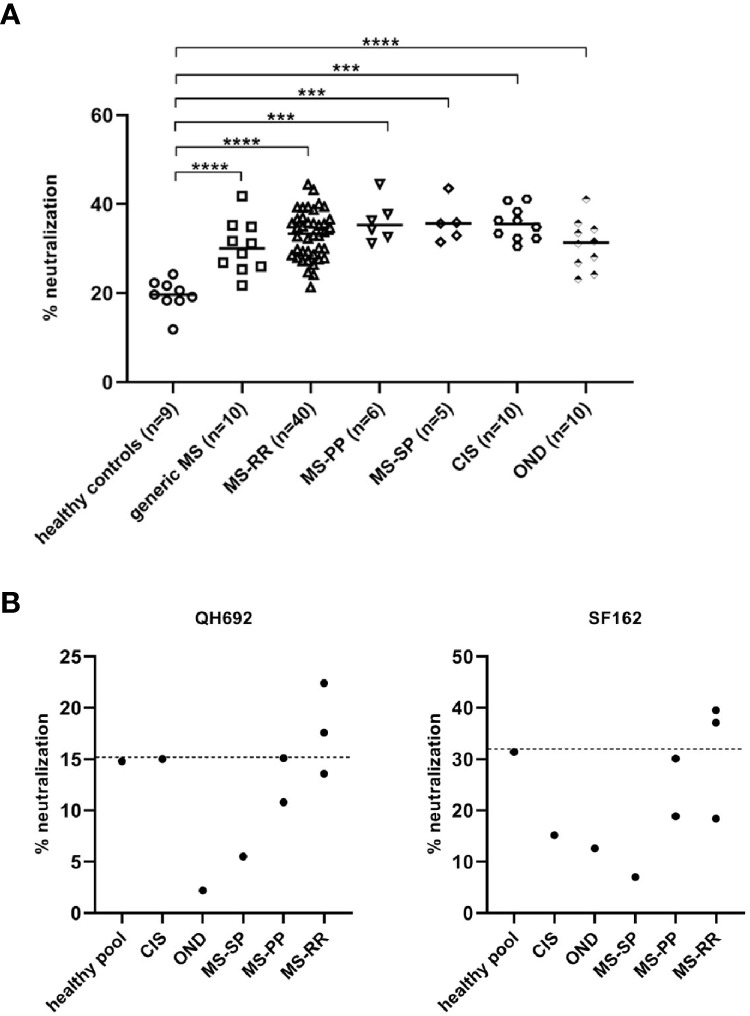
**(A)** HIV-QH692 pseudovirus-blocking activity of MS-RR patients (n = 40), MS-PP (n = 6), MS-SP (n = 5), CIS (n = 10) and OND (n = 10). Healthy donors were used as negative controls (n = 9). **(B)** HIV-QH692 or **(C)** HIV-SF162 pseudovirus-blocking activities of IgG fractions from CIS (n = 1), MS-RR (n = 3), MS-PP (n = 2), MS-SP (n = 1), OND (n = 1). p-values were calculated using Student’s T test (****p < 0,0001; ***p < 0,001).

Based on previously published findings (1), we wondered whether the production of bnAbs was restricted to SLE patients and its relative mouse models, or bnAbs were developed in other autoimmune diseases as well, such as MS. To this aim, were tested to investigate their inhibitory activity on the neutralization-resistant tier 2 virus HIV-QH0692 strain. The neutralization (NT) activity of the sera derived from patients with MS, although weak, was significantly higher than healthy controls. No difference in the percentage of neutralization among the different MS forms and patients with other neurological diseases has been observed, probably due to some soluble factors present in the sera of all patients tested (**Figure 1A**). In order to verify whether the neutralization activity was due to immunoglobulin (Igs) fractions rather than soluble factors, the Igs of the 8 sera showing the higher % NT (1 CIS, 3 MS-RR, 2 MS-PP, 1 MS-SP, 1 OND) were further purified. Only 2 sera from MS-RR patients confirmed the previously observed neutralizing activity, indicating that it was specifically due to Igs (**Figure 1B**). Interestingly, these patients were not experiencing a relapse at the moment of the sampling, suggesting that no inflammatory responses, with the consequent release of chemokine or cytokines, were involved in the block of the pseudovirus infection.

### EAE Mice Do Not Mount a gp140-specific IgG Response

After confirming that a small number of patients with MS displayed IgG-specific HIV-1 neutralizing activity in sera, we investigated the role of autoimmunity on the production of anti–HIV-1 neutralizing antibodies. We tested, therefore, the possibility to elicit such Abs at high titres in the MS mouse model EAE.

**Figure 2:**
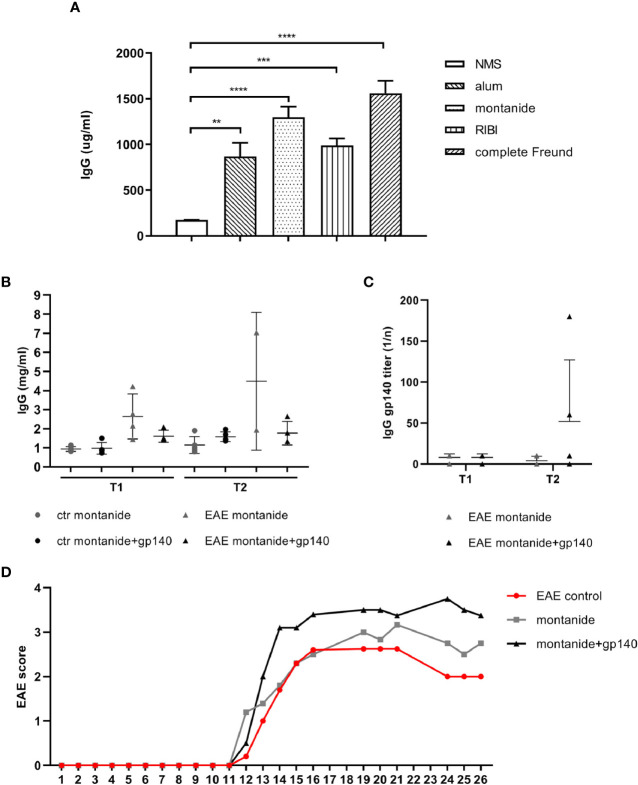
**(A)** Total IgGs serum concentrations obtained with 4 different adjuvants in C57BL/6 mice (n = 5). C57BL/6 healthy or EAE mice were immunized with montanide alone (n = 5) or in combination with gp140 (n = 5). Total IgGs serum concentration expressed as mg/ml **(B)** and gp140-specific IgG titers expressed as 1/n **(C)** measured by ELISA are shown at 7 days p.i. (T1) and 14 days p.i. (T2). **(D)** Clinical evaluation of the EAE score in all experimental groups. All data are reported as the arithmetic mean ± SEM. p-values were calculated using the one-way Anova test (**p < 0.01; ***p < 0.001; ****p < 0.0001).

We compared four different adjuvants to assess their efficacies in terms of IgGs yield and safety profiles: Freund’s adjuvant, aluminum hydroxide, RIBI, and Montanide ISA 720. Freund’s adjuvant induces systemic humoral and cellular immunity, but it can often cause chronic inflammation and tissue necrosis at the injection site; similar to Freund’s adjuvant but less aggressive is montanide ISA 720, a stable oil-water emulsion; aluminium, widely used as adjuvant for its overall safety, generates mild inflammatory responses and achieves immune memory; RIBI induces the presentation of hydrophobic antigenic epitopes and generates humoral responses to epitopes in their native conformation (19). Based on the comparative safety profiles and efficacies on the induction of IgGs between adjuvants, montanide was chosen for further experiments (**Figure 2A**).

We evaluated the primary anti-Env antibody response in 28 weeks-old control and EAE C57BL/6 mice. Control and EAE C57BL/6 mice were immunized with montanide alone or together with the trimeric gp140 to produce specific anti-HIV Abs. Trimeric gp140 represents the ectodomain of Env, composed by the entire gp120 and 20 KDa of gp41, and it is used as immunogen to induce a more potent humoral immune response against HIV-1 compared to the monomeric gp120, since it mimics native Env on the virion (20).

We first measured the amount of total IgGs in serum at 7 (T1) and 14 (T2) days post immunization (p.i.). Immunization with gp140 did not show substantial differences compared to montanide alone on the amount of total IgGs in both control mice and EAE mice at any experimental time (**Figure 2B**). As observed for the total amount of IgGs, neither control mice nor EAE mice mounted a statistically significant specific anti-gp140 IgG response at any experimental time point (**Figure 2C**). Taken together, these results suggest that the severity of the disease status we have registered (**Figure 2D**) might affect the elicitation of the humoral immune responses in EAE mice against the gp140 antigen.

### Immunization of EAE Mice With Adjuvant Alone or in Combination With gp140 Do Not Induce Antibodies That Neutralize Tier 1 and 2 HIV-1 Strains

**Figure 3:**
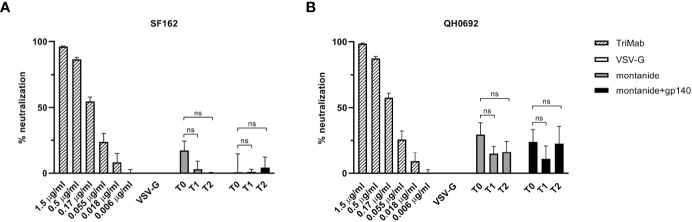
HIV-SF162 **(A)** and HIV-QH0692 **(B)** pseudoviruses-blocking activities of C57BL/6 B6 EAE mice 7 (T1) and 14 (T2) days after immunization with montanide alone or in combination with gp140, compared to time before immunization (T0). All p-values were calculated using Student’s t test assuming unequal variance (ns, not significant).

We next asked whether the Env-specific antibody response elicited in EAE mice (although not statistically significant, as shown in **Figure 2C**) was able to neutralize HIV-1 using a standardized *in vitro* TZMbl assay. Serum neutralization was tested against two HIV-1 clade B viruses representing the neutralization-sensitive tier 1 (SF162) and neutralization-resistant tier 2 virus strains (QH0692). As showed in **Figure 3**, none of these sera displayed neutralizing activity against neither the SF162 strain nor the QH0692 strain, indicating that gp140 Abs elicited in control mice did not contribute to neutralization.

### Gp140 Specific Abs Are Not Elicited in EAE Mice and Do Not Show Any Neutralization Activity

In order to investigate if the negative correlation between HIV-1 and MS might be related to the presence of bnAbs, we attempted at inducing the development of such Abs in mice to study their potential effect on the onset of the EAE or its clinical course.

**Figure 4:**
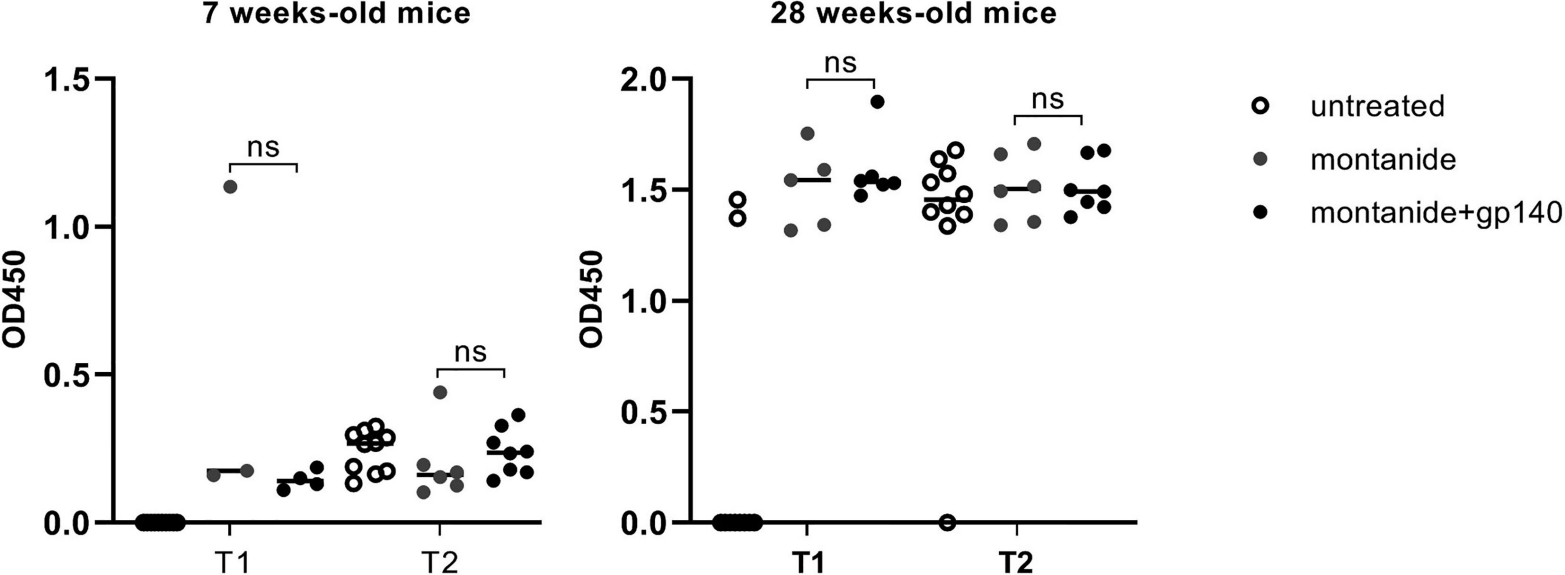
Gp140-specific IgG titers are shown at 7 days p.i. (T1) and 14 days p.i. (T2) in untreated, montanide or montanide+gp140-treated 7 weeks-old and 28 weeks-old C57BL/6 EAE mice. p-values were calculated using two-way ANOVA test (ns, not significant).

To this aim, 7 weeks and 28 weeks-old C57BL/6 EAE mice were immunized with montanide alone or together with the trimeric gp140 to elicit specific anti-HIV Abs and gp-140-specific Abs were measured 7 and 14 days p.i. by ELISA. As showed in **Figure 4**, no statistically significant difference in terms of gp140-specific Abs production was measured neither 7 days p.i. nor 14 days p.i., confirming the challenges of the induction of anti-HIV Abs through vaccination.

**Figure 5:**
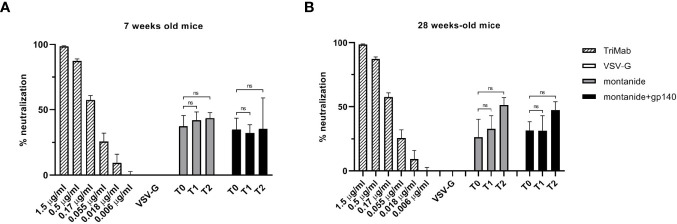
HIV-QH0692 pseudoviruses-blocking activity of 7 **(A)** and 28 weeks-old C57BL/6 mice **(B)** before immunization (T0), 7 days (T1) and 14 days (T2) after immunization with montanide alone or in combination with gp140. NS, not significant.

To further analyze the sera of immunized mice, we evaluated their neutralization activity on the HIV-QH0692 strain. As represented in **Figure 5**, neither 7 weeks-old mice sera (**Figure 5A**) nor 28 weeks-old mice sera (**Figure 5B**) showed any statistically significant neutralizing activity after immunization with adjuvant alone or in combination with gp140.

### Immunization With gp140 Does Not Affect the Onset of EAE

**Figure 6:**
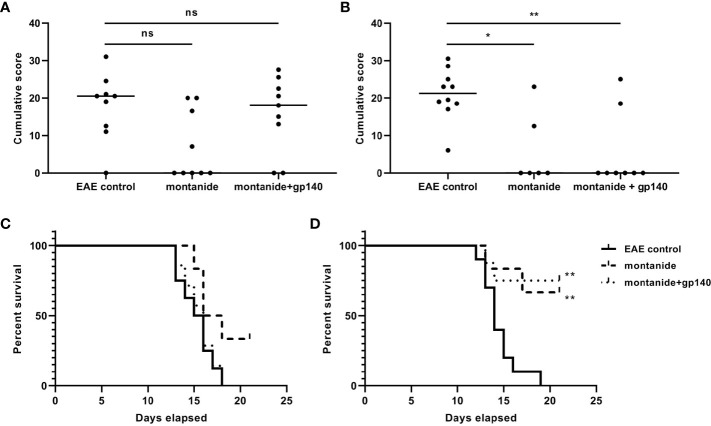
Clinical evaluation of EAE. Score of the disease in 28-weeks **(A)** and 7-weeks old C57B/6 mice **(B)**. The data are plotted as the mean daily clinical score for all animals in a particular treatment group. Kaplan–Meier analysis showing the statistically significant differences in the median time of EAE onset in 28-weeks **(C)** and 7-weeks old C57B/6 mice **(D)**. Significance is expressed as follows: *p < 0.05, **p < 0.01, ns, not significant.

We wanted to verify the effect of the immunization of onset and/or the clinical course of EAE. To this aim, EAE was induced in 7- and 28-weeks old C57BL/6 mice 14 days after the immunization with gp140. The experimental groups composition of mice was as follows: group 1: 10 untreated C57BL/6 mice (healthy controls); group 2: 10 untreated C57BL/6 mice (EAE controls); group 3: 10 C57BL/6 mice receiving montanide ISA 720 and EAE induction; group 4: 10 C57BL/6 mice receiving gp140 and EAE induction. Mice were weighed and scored for clinical signs daily up to the day of culling. As shown in **Figure 6**, the disease was less severe in 7-weeks old mice immunized with gp140 compared to untreated mice, but no difference was observed between those mice receiving gp140 and those receiving the adjuvant alone (**Figure 6A**). The same phenotype was observed for mice weights (**Figure 6C**). No differences neither in terms of disease severity nor mice weight was observed in 28 weeks-old mice immunized with gp140 (**Figures 6B, D**). Taken together, these results showed that gp140 immunization did not have any effect on the clinical manifestations of EAE, probably due to the inefficiency in gp-140-specific Abs elicitation.

## Discussion

The characterization of a subset of Env-specific bNAbs with autoreactive properties (5, 6, 22–24) raised the hypothesis that tolerance may restrict Env-specific autoreactive B cells from mounting an HIV-1 antibody response. Normally, autoreactive B cells are subjected to negative selection in the bone marrow, where they are eliminated or silenced by mechanisms of central tolerance. However, some weak autoreactive B cells escape from the central B cell tolerance, enter the peripheral lymphoid compartments of healthy individuals and wild-type mice and become anergic (25–28). A study carried out by Schroeder and collaborators confirmed that immunological tolerance impaired B cells from producing Env-specific antibodies able to neutralize tier 2 HIV-1 strains. They also demonstrated that breaching tolerance led to the production of HIV-1–neutralizing antibodies in mice with wild-type immune systems (8). Previous studies revealed that patients with SLE (13–15) and autoimmune prone mice (11, 12) exhibited Env specific bNAbs, even in the absence of infection. Of note, a SLE patient harbored plasma able to neutralize a wide breadth of HIV-1 strains and controlled HIV-1 infection in the absence of antiretroviral therapy (5). As reported for SLE (13, 16, 17), a negative correlation between HIV-1 infection and MS has been observed (29). Accordingly, very few cases of comorbidity of MS and HIV-1 have been reported (30); however, to date, it remains unclear. MS lesions are characterized by immune infiltrations due the presence of CD4+ and especially CD8+ T cells, B cells and other immune cells that play major roles in the pathophysiology of MS (31). Therefore, the immunosuppression caused by the chronic HIV-1 infection itself, leading to a progressive loss of CD4+ T lymphocytes, might prevent the development of MS in HIV-1 infected subjects.

So far, no information about Env specific reactivities have been reported in MS patients. To our knowledge, we showed for the first time that a small number of HIV-1 negative, relapsing remitting MS patients exhibited antibodies with neutralizing properties. A limitation of this study is the limited number of healthy subjects included in the analysis. This finding let us to better characterize the mechanisms by which the peripheral tolerance might limit B cells to mount neutralizing Env-specific antibody responses using the EAE mouse model of multiple sclerosis.

Whereas the study of Schoroeder and colleagues (8) reported that both healthy wild-type and lupus- prone mice mount Env-specific antibody responses, but only the latter have neutralizing activity, in our model of EAE we did not observed a statistically significant induction of such antibodies. In addition, in contrast with the lupus mice models, we did not observed any difference between younger and older mice, whereas high titers of autoreactive antibodies were observed in MRL/lpr mice who develop lupus disease at younger ages than B6.Sle123 mice (10).

Priming and activation of appropriate B cell precursors for HIV bnAbs seems to be a major obstacle to the elicitation of bnAb responses in humans (32). It has been demonstrated that Env does not efficiently engage naive B cells expressing germline B-cell antigen receptors (BCRs) of bNAbs; therefore, the process of B cell maturation that would lead to the production of bNAbs do not take place (33, 34). Interestingly, Steichen and collaborators (35) designed a successful germline-targeting immunogen with affinity for a pool of potential bnAb precursors. This immunogen induced bnAb-precursor B cell responses that potentially mature into bnAbs (35). Priming conditions are crucial for the breadth and the durability of the B cell response, and we cannot exclude that in the EAE model we should evaluate a more efficient engineered immunogen for priming the induction of HIV bnAbs.

Many subsets of T cells cooperate in the onset, maintenance and recovery of EAE, including T-helper-type 17 cells and regulatory T cells. Together with a strong T-cell mediated response, B cells producing demyelinating antibodies and macrophages are crucial effectors cells in EAE initiation. Inflammation plays a key role in the pathogenesis of MS (36). IFN-γ– and IL-17–producing T cell subsets are important for promoting EAE (37, 38), whereas a rare population of IL-10-producing regulatory B cells are involved in suppressing the onset of the disease. Importantly, the cytokines and growth factors present in the microenvironment in which B cell priming occurs, might affect the antibody response (39). In this study, we did not measure the levels of pro-inflammatory cytokines. Env immunization could not have modulate the hyperactivity of the immune system reverting the balance between pro- and anti-inflammatory cytokines in EAE mice, or perhaps it could have enhanced their production exacerbating the clinical course of the disease. A more detailed analysis of the molecular composition of the sera after immunization might enable a more specific evaluation of the degree of efficiency of the immunization, which could improve the immunization study design. A recent paper showed that the administration of the Tat protein, one of the major player involved in immunosuppression, led to a marked decrease in the clinical score of EAE mice, as well as improvement in motor-neuron functions. EAE mice treated with HIV-1 Tat clade B and C exhibited significant improvement in the clinical motor signs throughout the disease course, by decreasing the levels of proinflammatory cytokines (40).

It has been demonstrated that Env could induce neurotoxicity in the presence of astrocytes and microglia cells that are upregulated after EAE induction. Kong et al. demonstrated that glia cells exposed to HIV-1IIIBgp120 released nitric oxide and the pro-inflammatory cytokines TNF-α and IL-6 in murine primary mixed glial cell cultures. The neurotoxic effects of gp120 were significantly enhanced by priming glial cells with IFN-γ (41). We can speculate that in our model of immunological tolerance breaking, Env immunization could have exacerbated the detrimental effects of EAE induction rather than ameliorating the status of the disease.

Collectively, we were not able to induce potent bnAbs in a different animal model of autoimmune disease. Our study has some limitations. The success in eliciting such antibodies might be related to the specific characteristics of the autoimmune disease, or it might rely in improving the vaccination design. Certainly, the balance between the immune cell subsets and their microenvironment, including soluble factors that modulate the outcome of the responses, needs to be taken into consideration when approaching an immunization strategy to overcome the challenges in bNAbs induction.

## Data Availability Statement

The original contributions presented in the study are included in the article/supplementary material. Further inquiries can be directed to the corresponding author.

## Ethics Statement

The studies involving human participants were reviewed and approved by San Raffaele Scientific Hospital Ethical Committee. The patients/participants provided their written informed consent to participate in this study. The animal study was reviewed and approved by Institutional Animal Care and Use Committee (IACUC), San Raffaele Scientific Institute (Milan, Italy).

## Author Contributions

GS, AF, and CP performed the biological tests and analysed the data. GS, AF, LL, and RF designed the experiments. VM provided clinical data. GS wrote the manuscript. LL: conceptualization. RF and LL revised the manuscript. All authors contributed to the article and approved the submitted version.

## Funding

This work has been supported by Fondazione Italiana Sclerosi Multipla – FISM grant 2016/R/15 to RF.

## Conflict of Interest

The authors declare that the research was conducted in the absence of any commercial or financial relationships that could be construed as a potential conflict of interest.

## Publisher’s Note

All claims expressed in this article are solely those of the authors and do not necessarily represent those of their affiliated organizations, or those of the publisher, the editors and the reviewers. Any product that may be evaluated in this article, or claim that may be made by its manufacturer, is not guaranteed or endorsed by the publisher.
